# Three-dimensional technology assisted trabecular metal cup and augments positioning in revision total hip arthroplasty with complex acetabular defects

**DOI:** 10.1186/s13018-019-1478-1

**Published:** 2019-12-11

**Authors:** Qingtian Li, Xuepan Chen, Bofu Lin, Yuanchen Ma, Jun Xing Liao, Qiujian Zheng

**Affiliations:** 1 Department of Orthopedics, Guangdong Provincial People’s Hospital, Guangdong Academy of Medical Sciences, School of Medicine, South China University of Technology, No. 106, Zhongshan Second Road, Yuexiu District, Guangzhou, 510000 China; 2 Shantou University Medical College, Shantou, China

**Keywords:** Acetabular defect, Revision total hip arthroplasty, Three-dimensional simulation, Three-dimensional model

## Abstract

**Background:**

Revision total hip arthroplasty (THA) with large acetabular defect remains a challenge. Though trabecular metal (TM) cup and augments have been introduced in defect reconstruction with good result, the accurate positioning of implant is important to avoid complications. Therefore, we aimed to evaluate the usefulness of three-dimensional (3D) simulation and 3D model in assisting implant positioning during complex revision THA.

**Methods:**

Sixteen patients (18 hips) who underwent revision THA with a Paprosky type III acetabular defect were analyzed retrospectively. Placement of acetabular cup and TM augments was simulated with 3D simulation software and 3D model preoperatively. Cup anteversion, abduction angle, and hip center were measured in each case preoperatively and postoperatively. Primary outcome was the percentage of outliers according to Lewinnek safe zone and Harris hip score (HHS). Secondary outcome was the correlation between the 3D planned and the postoperative value.

**Results:**

The percentage of outliers was significantly corrected from 77.78% (14/18) preoperatively to 38.88% (7/18) postoperatively (*p* = 0.04). There was a significant correlation between mean planned cup anteversion and postoperative value (13.39 vs 11.99, *r* = 0.894; *p* < 0.001). There was a significant correlation between mean planned abduction and postoperative value (42.67 vs 44.91, *r* = 0.921, *p* < 0.001). The number of planned and used augments was the same in all the cases. In 15 cases (83.33%), the size of planned and used TM augments was the same. The HHS was significantly improved at final follow-up (80.94 vs 27.50, *p* < 0.001). No cases presented dislocation or radiological signs of loosening.

**Conclusion:**

Preoperative 3D simulation and model were considered the useful method to assist implant positioning in revision THA with complex acetabular defect, with moderate to high accuracy and satisfied clinical outcome.

## Background

As the aging population is coming, more and more primary total hip arthroplasty (THA) will be performed annually [1]. As a result, the revision THA is estimated to increase exponentially in the future. Compared with primary THA, the revision THA is more difficult with complications, such as the intraoperative fracture, dislocation risk, and aseptic loosening [2, 3].

In revision THA, acetabular defect is not uncommon and regarded as a challenge. Normally, the type of bone loss and remaining bone quality determine the way of acetabular reconstruction [4]. The trabecular metal cups and augments have been commonly used together for individualized acetabular defect reconstruction with satisfied outcome [5, 6]. Characteristics of TM implant including high porosity and low modulus of elasticity could provide strong primary stability and promote a deep bony in-growth [7].

The outcome of revision surgery strongly depends on the accurate position of implant [8]. Malposition of the acetabular component increases the dislocation risk and other complications [9]. Previous study showed preoperative 3D planning technology improved the accuracy of implant position in primary THA [10]. However, application of 3D simulation and model to assist TM cup and augments positioning in revision THA was rarely reported [11, 12]. We aimed to evaluate the advantages and disadvantages of this technology and to provide suggestions for its clinical application in revision THA with complex acetabular defect.

## Patients and methods

### Patient population

Ethics approval was obtained from our hospital institutional review board. We performed a retrospective study using our hospital electronical medical records, including clinical notes, imaging, and clinical scores. We identified all patients who underwent revision THA with using the 3D simulation plan and 3D model from May 2013 to July 2017.

The inclusion criteria are as follows: (1) revision THA with acetabular defect, Paprosky type III; (2) using both TM cup and augments in acetabulum reconstruction; (3) using preoperative 3D simulation plan and 3D model based on the preoperative computed tomography (CT) scans.

The exclusion criteria are as follows: (1) primary THA; (2) the reasons of first THA were tumor or infection.

A total of 16 patients (18 hips) undergoing acetabular revision procedures were included in the study.

### Preoperative planning protocol

#### Three-dimensional pelvis model

Before the surgery, patients underwent computer tomography (CT) scan. Scans covered the pelvis and femur with a 1.25-mm slice thickness (General Electric Company, USA). The CT scans were performed at the same medical imaging center with the same parameters.

Then, the CT scan data was imported into radiological post-processing software Materialise Mimics software (version 15.0; Materialize, Leuven, Belgium) to create a simulated model of the pelvis. After reconstruction, the 3D images of pelvis were simultaneously displayed in software and data was transported to the 3D printer (Formlab, America); then, 3D pelvis model was printed using the resin material. Finally, we could get a 3D pelvis model in real size with all defects, which helped to assess the acetabular bone defect. In our study, we classified the acetabular defect according to Paprosky classification [13].

#### Simulation of cup and TM augments positioning

**Fig. 1:**
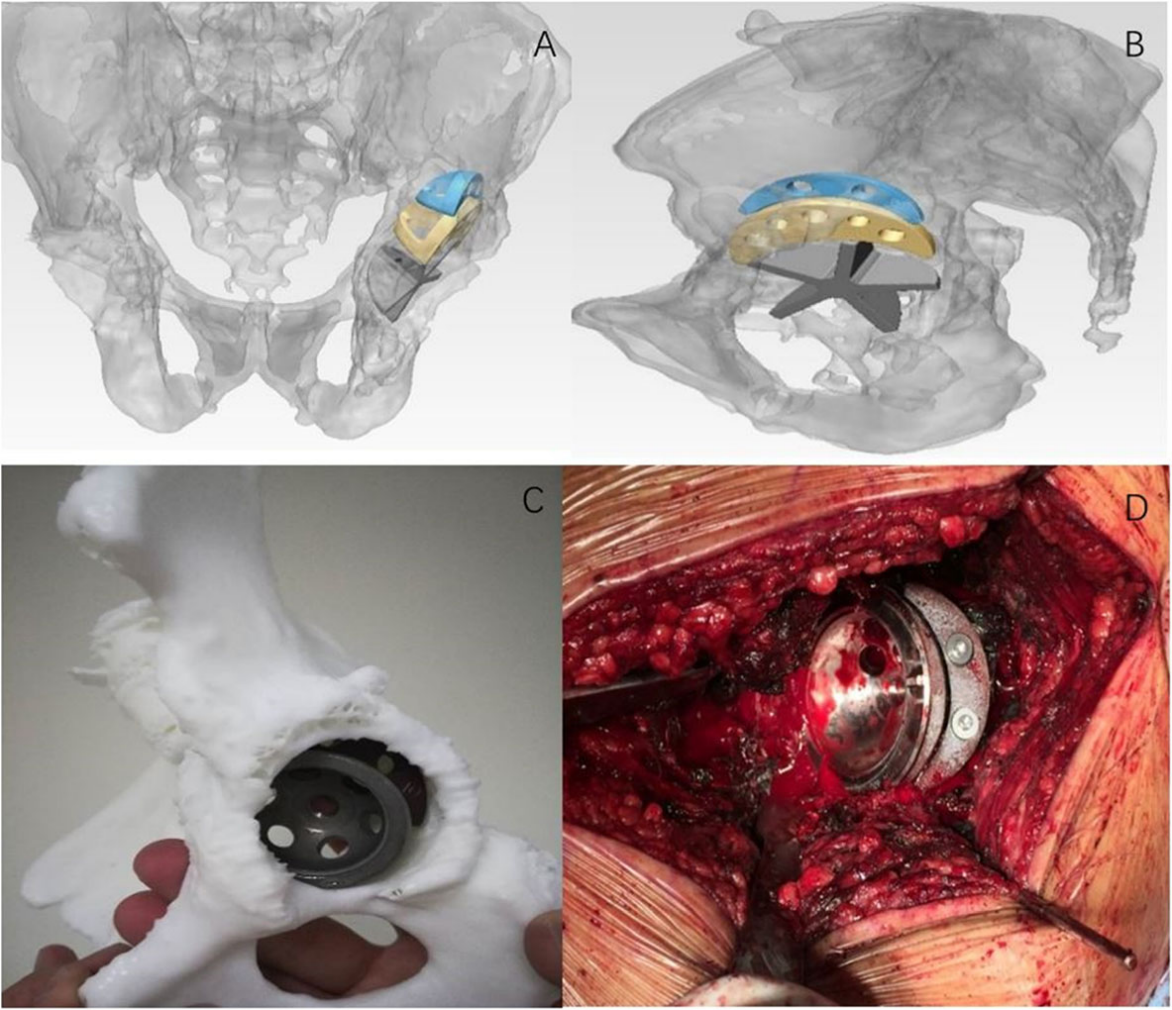
**a**, **b** Cup and trabecular metal augments positioning with 3D simulation technology. **c** Cup and trabecular metal augments were positioned in 3D plaster model. **d** Cup and trabecular augments were placed based on the 3D plan

The acetabular cup and TM augment positioning process were simulated in Materialise Mimics software. The acetabular cup (Zimmer, USA) and TM augment templates (Zimmer, USA) were placed in 3D virtual acetabulum step by step (Fig. 1a, b). The cup orientation was determined by preoperatively set abduction and anteversion angles. The distance from the cup edge to acetabular edge was measured relative to the anterior, posterior walls as well as to the superior edge in software, which was given to surgeons in operation. In addition, the plan of 3D simulation was rechecked by positioning cup and augments in pelvis model again (Fig. 1c). If it was proved to be practicable and suitable, the final views of the cup and trabecular augments within the acetabulum were given to the surgeon at the time of surgery.

### Surgical protocol

The 3D printed pelvis model was sterilized and prepared for using in surgery. The revision THA was performed by the same medical team using a posterolateral approach. First of all, we removed the previous failed hip implant, debrided the remaining acetabular cavity, and reassessed acetabula bony defect comparing with the 3D pelvis model. The acetabulum was gently reamed until contact was made with the bleeding host bone, then inserted the new cup and TM augments based on the parameters from preoperative 3D planning (Fig. 1d). We tried to make the distance from cup edge to acetabular edge as the same as the values of 3D planning by using sterilized flexible ruler in operation.

### Rehabilitation after operation

On the first day after surgery, patients were encouraged to move the knee along with static quadriceps. Partial weight-bearing walking with crutches was requested until 6 weeks after surgery; a full weight-bearing gait was permitted at 6 weeks postoperatively.

### Parameters on X-ray

**Fig. 2:**
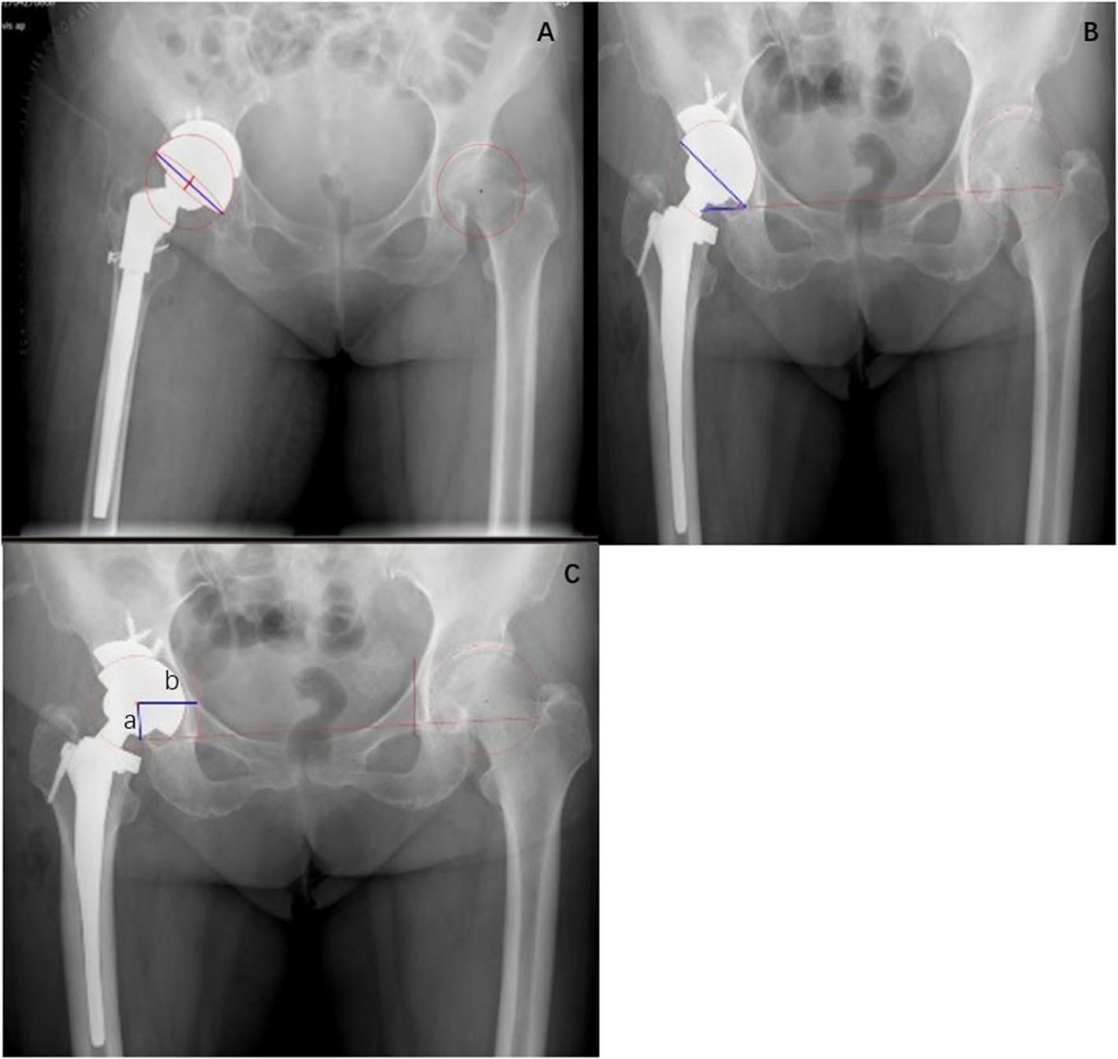
Measurement with anteroposterior view of an X-ray. **a** measures the angle of anteversion, anteversion angle = arcsin (D1/D2); D1: short red line of the ellipse; D2: long blue line. **b** measures the angle of abduction. **c** shows the vertical and horizontal position of COR. A: vertical position of COR; B: horizontal position of COR

In all patients, preoperative and postoperative angle measurements were performed on standardized radiographs of the Picture Archiving and Communication System (PACS). The radiographs comprised an anteroposterior view of the pelvis centered over the pubic symphysis with the hips at 15° of internal rotation. The instructions of parameter are shown in Fig. 2.

Inter- and intra-observer reliability was assessed by intraclass correlation coefficients (ICCs) for measurements of angle parameters. For the interobserver reliability, all the angle parameters were measured by two independent authors (X.P.C; Q.T.L). For the intra-observer reliability, the same radiograph was measured by each observer after 1 month. The ICCs larger than 0.8 were considered to have excellent reliability of measurement. Excellent reliability of measurement was found in our study (ESM-1), so radiological measurements from only one investigator were included in analysis.

### Acetabular cup anteversion angle

We measure the acetabular cup anteversion angle on anterior-posterior pelvic plain radiograph. According to Lewinnek, anteversion angle = arcsin (D1/D2) [14]. D1 is the distance of the short axis of the ellipse; D2 is the long axis of the acetabular component, reflecting the maximum diameter of the cup (Fig. 2a).

### Acetabular cup abduction angle

The abduction angle was the angle between acetabular axis and trans-ischial in the coronal plane (Fig. 2b).

### Position of center of rotation

The center of rotation of hip was confirmed by using a concentric circular region of interest that was digitally drawn to best fit the acetabular cup. Vertical position of the center of rotation (COR) was defined as the distance between the COR and the inter-teardrop line. The horizontal position of COR was defined as the distance from the COR to the floor of the acetabular teardrop (Fig. 2c).

### Safe zone

The “safe zone” for acetabular inclination and anteversion in total hip arthroplasty (THA) was firstly defined by Lewinnek et al. in 1978 [14]. They defined the ideal inclination angle of acetabular cup was 30° to 50°, and the optimal anteversion angle was 5° to 25°.

### Evaluation criteria

The main evaluation criterion was the percentage of postoperative outliers according to the Lewinnek safe zone. The second criterion was the correlation between the preoperative plan and the postoperative measurement. The third criterion was the difference between the number and size of planned and postoperative used metal augments. Patients were assessed clinically according to the Harris hip score before surgery and at final follow-up.

### Statistical analysis

Kolmogorov-Smirnov test was used for testing normality. Data of abduction angle was non-normally distributed; data of anteversion angle, hip center, and HHS was normally distributed. Paired *T* test was used to assess the difference between preoperative/postoperative; 3D planned/postoperative anteversion angle. Wilcoxon test was used for abduction angle. Chi-square test was used for comparing preoperative/postoperative percentage of outliers. The Pearson coefficient test was used to assess the correlation between planned and postoperative anteversion angle and Spearman test for assessing correlation of abduction angle. Statistical significance was defined as a two-tailed *p* value < 0.05. The analysis was performed using SPSS 20 (Chicago, IL, USA).

## Results

### Demographic data

From May 2013 to July 2017, 18 patients (20 hips) underwent acetabular reconstruction using trabecular metal cup and augments with 3D preoperative planning. After reviewing medical records, 2 patients were excluded due to primary THA. Finally, 16 patients (18 hips) were included in the study and the demographic data is demonstrated in Table 1.

**Table 1:** Patient characteristics

Case number	Age (years)	Gender	Primary diagnosis	Years since first replacement	Reason for revision	Paprosky classification	Surgery side	Follow-up (months)	Preoperative HHS	Postoperative HHS
1	60	Male	AVN	12	Aseptic loosening	3B	L	53	26	86
2	52	Male	AF	18	Aseptic loosening	3A	L	45	20	71
3	52	Female	AVN	15	Aseptic loosening	3A	R	45	21	84
4	44	Male	FNF	6	Septic loosening	3A	R	41	20	80
5	60	Male	AVN	21	Aseptic loosening	3A	L	34	17	69
6	45	Female	AVN	9	Aseptic loosening	3A	R	34	18	82
7	62	Male	AVN	20	Aseptic loosening	3A	R	31	30	85
8	67	Female	FNF	20	Aseptic loosening	3A	L	30	25	77
9	58	Male	OA	10	Aseptic loosening	3A	R	27	32	80
10	68	Male	FNF	10	Septic loosening	3B	L	24	35	87
11	59	Female	OA	8	Aseptic loosening	3A	R	18	28	79
12	68	Male	AVN	22	Aseptic loosening	3B	L	17	25	89
13	54	Male	AVN	10	Septic loosening	3B	R	14	32	84
14	69	Male	AVN	30	Aseptic loosening	3A	L	21	28	81
15	69	Male	AVN	30	Aseptic loosening	3A	R	21	28	81
16	49	Male	AS	25	Aseptic loosening	3A	L	14	36	83
17	49	Male	AS	25	Aseptic loosening	3B	R	14	36	83
18	60	Female	OA	11	Aseptic loosening	3A	L	16	32	76

*AVN*, avascular necrosis of femoral head; *L*, left; *R*, right; *AF*, acetabular fracture; *FNF*, femoral neck fracture; *OA*, osteoarthritis; *AS*, ankylosing spondylitis, *HHS*, Harris hip score

Patients included 11 men and 5 women, with mean age of 58.06 ± 8.29 years at the time of surgery. Paprosky type IIIA defect was found in 13 hips, whereas a Paprosky type IIIB defect was present in 5 hips. The reasons for the primary THA were hip osteoarthritis (*n* = 3), hip fractures (*n* = 4), avascular necrosis of femoral head (*n* = 9), and ankylosing spondylitis (*n* = 2). The mean time from the primary THA to the revision was 16.78 ± 7.46 years. The etiology requiring revision THA was aseptic loosening in 15 hips and septic loosening in 3 hips.

### Intraoperative data

The mean operation time was 254 ± 91 min; blood loss was 891 ± 423 ml. The mean blood transfusion was 860 ± 400 ml.

### Radiologic outcome

**Fig. 3:**
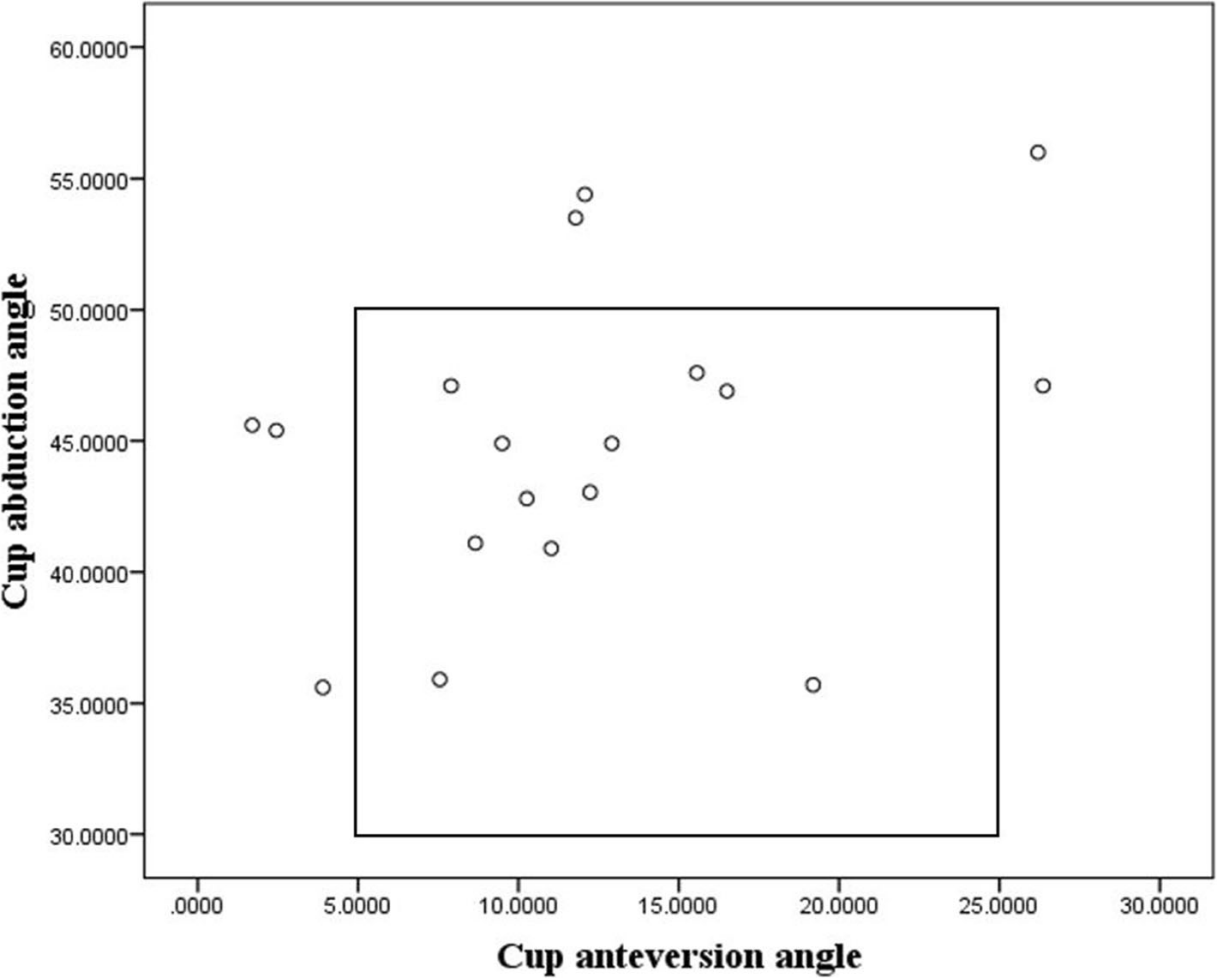
Scatter plot of each hip’s abduction and anteversion angles within the safe zone of Lewinnek et al.

Based on the Lewinnek safe zone, 11 of 18 (61.1%) patients were positioned within the safe zone (Fig. 3). Of the 7 outliers, 3 were out of the abduction safe zone, 5 were out of the anteversion safe zone, and 1 was out of both safe zones. Percentage of outliers was corrected from 77.78% (14/18) preoperatively to 38.89% (7/18) postoperatively, with statistical significance (*p* = 0.040).

As shown in Table 2, ratio of vertical position of COR in surgical site/contralateral site was corrected from 1.15 ± 0.19 to 1.09 ± 0.20 postoperatively (*p* = 0.185). Ratio of horizontal position of COR in surgical site/contralateral site was changed from 0.97 ± 0.21 to 1.00 ± 0.18 postoperatively (*p* = 0.193). Though the position of COR was corrected to be nearly as the same as contralateral site, no statistical significance was found in our study.

**Table 2:** Anteversion, abduction, position of COR before and after revision THA

Parameters	Preoperative	Postoperative	3D planned	Postoperative-preoperative value; *p* value	Postoperative-planned value; *p* value
Anteversion	11.35 ± 8.55	11.99 ± 6.91	13.39 ± 3.48	0.06 ± 12.44; *p* = 0.982	− 1.39 ± 4.1; *p* = 0.168
Abduction	59.60 ± 31.15	44.91 ± 5.93	42.67 ± 4.40	− 13.92 ± 32.90; *p* = 0.090	2.24 ± 3.02; *p* = 0.006
Vertical position of COR/contralateral position of COR	1.15 ± 0.19	1.09 ± 0.20	/	− 0.44 ± 1.38; *p* = 0.185	
Horizontal position of COR/contralateral position of COR	0.97 ± 0.21	1.00 ± 0.18	/	0.06 ± 0.19; *p* = 0.193	/
HHS	27.50 ± 6.54	80.94 ± 5.19	/	53.44 ± 6.40; *p* < 0.001	/

*COR*, center of rotation; *THA*, total hip arthroplasty; *HHS*, Harris hip score

**Fig. 4:**
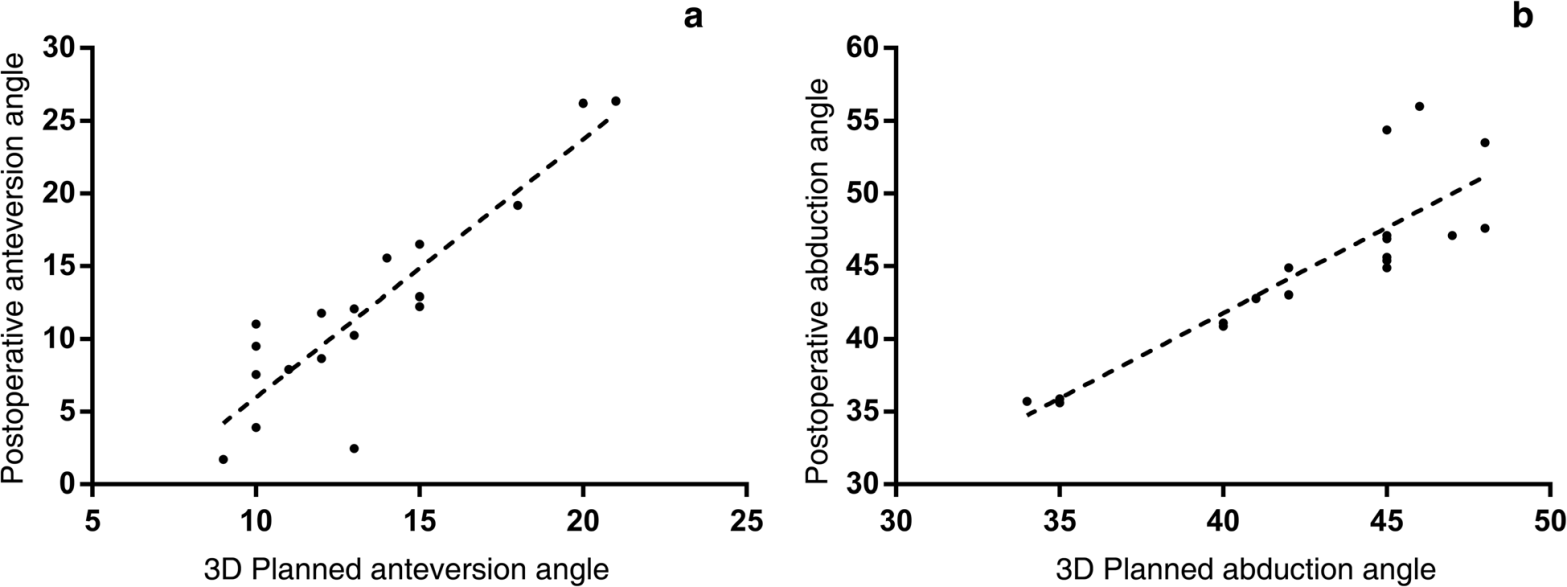
**a**, **b** There was a significant correlation between the planned and the final postoperative values of cup anteversion and abduction

The correlation between planned and postoperative cup orientation is shown in Fig. 4. The mean planned cup anteversion value did not differ from the postoperative value (− 1.39 ± 4.1; *p* = 0.168), and a strong correlation was found (*r* = 0.894; *p* < 0.001). There was deviation between the mean planned abduction and the postoperative value (2.24 ± 3.02; *p* = 0.006), but a strong correlation between these two values was found (*r* = 0.921, *p* < 0.001).

Number and size of 3D planned and used TM augments are shown in Table 3. Thus, the number of planned and used augments was same in all the cases. In 15 cases (83.33%), the size of planned and used TM augments was the same. In other cases, the difference was not over 4 mm.

**Table 3:** Comparison of number and size of planned and used augments

Case number	3D planned		Used	
	Number	Size (diameter × thickness)	Number	Size (diameter × thickness)
1	2	50 × 15, 50 × 10	2	50 × 15, 50 × 10
2	1	54 × 10	1	54 × 10
3	1	50 × 10	1	50 × 10
4	1	54 × 10	1	54 × 10
5	1	58 × 10	1	58 × 10
6	1	54 × 10	1	54 × 10
7	1	50 × 20*	1	54 × 20
8	1	54 × 10	1	54 × 10
9	1	54 × 10	1	54 × 10
10	2	50 × 20, 50 × 10*	2	50 × 20, 50 × 15*
11	2	54 × 10, 50 × 10	2	54 × 10, 50 × 10
12	2	50 × 10, 54 × 20	2	50 × 10, 54 × 20
13	3	54 × 10, 58 × 10*, 54 × 20	3	54 × 10, 54 × 10*, 54 × 20
14	1	54 × 10	1	54 × 10
15	1	54 × 10	1	54 × 10
16	1	54 × 10	1	54 × 10
17	1	54 × 10	1	54 × 10
18	1	50 × 15	1	50 × 15

### Clinical outcome

Follow-up data were available for all the patients included with a mean length of follow-up of 27.72 ± 12.18 months. The mean HHS was significantly improved from 27.50 ± 6.54 preoperatively to 80.94 ± 5.19 at final follow-up (*p* < 0.001). To sum up, the final scores were excellent in 0 hip, good in 13 hips, modest in 4 hips, and poor in 1 hip. The patient with lowest score was mainly influenced by the non-surgical site, which was also aseptic loosening but without surgery.

### Complications

Two cases had intraoperative periprosthetic femoral fracture, which were both fixed by locking plates and proceeded to bony union at final follow-up. There was no dislocation or revision required in any cases. There was no migration of acetabular component or loosening of the TM augment.

## Discussion

For complex revision THA with large acetabular defect, loss of normal anatomic landmarks increases the difficulty of implant positioning. An optimal implant position can prevent many complications, such as dislocation, acetabular component migration, wear, and osteolysis which affect the overall clinical outcome [15].

The type of implant used for acetabular defect reconstruction was variable in literature, such as reconstruction rings with structural allograft [16], cup cage reconstruction [17], and trabecular metal cups and augments [18]. The use of trabecular cup and TM augments maximized the contact with the host bone regardless of the size and shape of the bone defect, which was proved to have satisfied clinical outcome in previous studies [18, 19]. This study proved that using TM cup and augments had encouraging short- to mid-term outcomes. There was no sign of loosening or implant failure at final follow-up. Furthermore, there was high accuracy in predicting the number and size of used augments with 3D technique, which would greatly decrease the preoperative preparing time and provide valuable plan for the surgeons.

Restoring the COR to an anatomic position is important to hip biomechanics and implant stability [20]. Using cup and TM augments allows the surgeon to achieve a reduction of the migration of hip COR. In our study, we compared preoperative and postoperative ratio of position of COR in surgical site/contralateral site, which we wished the postoperative ratio to be more closed to 1. Both the vertical and horizontal positions of COR were corrected to be more similar with contralateral site, though there was no statistical significance, which may be due to the sample size.

Though the accuracy of implant position deeply influences the clinical outcome, the technique aiming to improve acetabular component position accuracy in revision THA was limited. For accuracy of freehand cup position in primary THA, Bosker et al. reported accuracy for cup placement in the Lewinnek safe zone were 85.2% and 82.7% in radiographic abduction and radiographic anteversion respectively [21]. Another study conducted by Minoda et al. also reported the accuracy of freehand cup position was over 80% [22]. However, freehand cup positioning has lower accuracy in revision THA. In a series case study involving 34 patients with Paprosky type III defects, only 19 (56%) were freehand positioned within the safe zone of Lewinnek postoperatively [23]. The ability of a surgeon to determine the difference between 15° and 30° was difficult and unreliable without the use of advanced technology [24]. Therefore, a supportive tool was needed to assist implant positioning in revision THA.

This study showed the 61.1% of revised cups were optimally positioned based on Lewinnek safe zone, which demonstrated good accuracy. In addition, 3D technology was also proved to be effective for primary THA in patients with complex acetabular deformity. Recently, Coral et al. reported a case of using 3D technology to assist primary THA in patient diagnosed with hip coxarthrosis due to previous untreated acetabular fracture (Paprosky type III). They found the center of rotation of the hip and length discrepancy was accurately recovered as preoperative plan without signs of loosening, subsidence, or osteolysis at final follow-up [25]. Compared with CT-based navigation, the 3D preoperative simulation technology and 3D model had lower accuracy [26–28]. Additionally, previous study reported there was a high accuracy by using imageless navigation-assisted cup positioning [29]. However, in contrast to CT-based navigation technique, the 3D planning was easy to achieve without increasing operative time and extra cost. Consequently, 3D planning was a good compromise method between accuracy on the one hand and extra cost and operative time on the other hand.

With the 3D simulation and pelvis model, preoperative 3D acetabula anatomy was assessed clearly, and the plan of cup and TM augments were known beforehand. This study found the strong correlation between final cup anteversion and abduction angles with the 3D planned value. There was deviation between the postoperative and 3D planned abduction value. This may be due to a poor exposure of the proximal part of the acetabulum, which influences measuring the distance from the cup to the superior edge. Another reason may be related to the change of patient position during surgery. All the cases in our study were in lateral supine position; the change of position may affect the pelvic tilt which influences the surgeon to assess the abduction angle.

There are several limitations of this study. Firstly, being a retrospective study design, it is never as ideal as randomized controlled trial for comparison of this technique with other technique of reconstruction. Secondly, this was a study with small but reasonable sample size, because the trabecular metal augments have limited indication in revision surgery. In the future, we plan to compare revision groups with or without 3D simulation and models.

## Conclusion

There was a statistically significant correlation between 3D planned and postoperative value. Preoperative 3D simulation and model were considered the useful method to assist implant positioning in complex revision THA, with moderate to high accuracy, and with satisfied clinical outcome and lower complication rate. Moreover, it had high accuracy in predicting number and size of TM augments used intraoperatively.

## Data Availability

The data and materials are available from the medical records department of Guangdong Provincial People’s Hospital

## Abbreviations

3D: Three-dimensional
COR: Center of rotation
HHS: Harris hip score
ICCs: Intraclass correlation coefficients
PACS: Picture Archiving and Communication System
THA: Total hip arthroplasty
TM: Trabecular metal
